# Autoantibodies to angiotensin-converting enzyme 2 in patients with connective tissue diseases

**DOI:** 10.1186/ar3012

**Published:** 2010-05-14

**Authors:** Yuko Takahashi, Shiori Haga, Yukihito Ishizaka, Akio Mimori

**Affiliations:** 1 Division of Rheumatic Diseases, Research Institute, International Medical Center of Japan, 1-21-1 Toyama, Shinjuku-ku, Tokyo 162-8655, Japan; 2 Department of Intractable Diseases, Research Institute, International Medical Center of Japan, 1-21-1 Toyama, Shinjuku-ku, Tokyo 162-8655, Japan

## Abstract

**Introduction:**

Angiotensin-converting enzyme (ACE) 2, a homolog of ACE, converts angiotensin (Ang) II into Ang(1-7), and the vasoprotective effects of Ang(1-7) have been documented. We explored the hypothesis that serum autoantibodies to ACE2 predispose patients with connective tissue diseases to constrictive vasculopathy, pulmonary arterial hypertension (PAH), or persistent digital ischemia.

**Methods:**

Serum was examined from 42 patients with systemic lupus erythematosus (SLE), scleroderma, or mixed connective tissue disease. Eighteen vasculopathy patients with PAH (five cases) and/or persistent digital ischemia (16 cases) were compared with 24 patients without these vasculopathies (control patients) for serum reactivity to purified recombinant human ACE2, using an ELISA.

**Results:**

The sera from 17 of the 18 (94%) vasculopathy patients had ELISA scores above the baseline level determined using control sera from 28 healthy subjects, and the mean ELISA score in the vasculopathy patients was significantly higher than that in the control patients (*P *< 0.0005). The relative activity of serum ACE2, which was defined using a reference serum, correlated inversely with the ELISA scores for serum anti-ACE2 antibodies in the 18 vasculopathy patients (*R*^2 ^= 0.6872). The IgG fraction from vasculopathy patients, but not from healthy subjects, inhibited ACE2 activities *in vitro*. Consistent with this, immunosuppressive therapy given to one SLE patient with digital necrosis markedly decreased the anti-ACE2 antibody titer and restored serum ACE2 activity.

**Conclusions:**

Autoantibodies to ACE2 may be associated with constrictive vasculopathies.

## Introduction

Angiotensin-converting enzyme (ACE) 2, a homolog of ACE, is a carboxypeptidase that degrades angiotensin (Ang) II to Ang(1-7) [1]. Ang(1-7) has vasodilating, antiproliferative, and antithrombotic properties that antagonize the action of Ang II and play vasoprotective roles [2-4]. Recent studies have demonstrated the therapeutic effects of ACE2 activation by a synthetic molecule [5] or of *ACE2 *gene transfer [6] in experimental pulmonary hypertension models.

Pulmonary arterial hypertension (PAH), a vasculopathy of unknown etiology, is a serious complication of connective tissue disease (CTD) [7]. One clinical study found reduced metabolism of ACE synthetic substrate in the pulmonary vascular bed of PAH-CTD patients, but not in primary PAH patients [8]. Persistent digital ischemia, which manifests as skin ulcers or necrotic lesions, is another intractable vasculopathy of CTD, and is strongly associated with Raynaud's phenomenon. A correlation between Raynaud's phenomenon and elevated systolic pulmonary arterial pressure has been reported in patients with systemic lupus erythematosus (SLE) [9]. PAH or persistent digital ischemia is less frequent than Raynaud's phenomenon, and these three vascular abnormalities are involved in CTD patients across different disease entities, including SLE, systemic sclerosis (SSc), and mixed connective tissue disease (MCTD).

Our preliminary examination suggested the presence of novel autoantibodies to ACE2 in the sera of two patients: a patient with SLE suffering from severe digital necrosis, and a patient with SSc accompanied by lethal PAH. Furthermore, the sera of the two patients lacked ACE2 activity. These findings prompted us to conduct the present study in order to explore the hypothesis that serum autoantibodies to ACE2 predispose patients with CTD to constrictive vasculopathies; that is, PAH and persistent digital ischemia.

## Materials and methods

### Study design

As many patients as possible among those with CTD and PAH or persistent digital ischemia (vasculopathy patients) in our hospital at time of the study were enrolled. Sera from these patients were studied in comparison with those from CTD patients without vasculopathy or from healthy subjects. The ethics committee of our hospital approved this study, and written informed consent was obtained from all patients and control subjects.

### Serum sampling

Fresh serum was obtained from all of the patients and normal subjects for the present study. Each serum sample was aliquoted to avoid repeated thawing and was stocked at -20°C until assayed.

### Diagnosis of connective tissue disease

Forty-two patients with SLE, SSc, or MCTD were studied. SLE was diagnosed according to the classification criteria of the American College of Rheumatology [10]. Patients with SSc met the classification criteria for the diffuse (n = 3) or limited (n = 6) form of SSc, as described in the literature [11]. Patients with MCTD met the criteria for MCTD from Kasukawa and colleagues [12] and the original definition of MCTD by Sharp and colleagues [13].

### Diagnosis of pulmonary arterial hypertension

PAH had been diagnosed in five patients with SSc, based on dyspnea on exertion, elevated plasma brain natriuretic peptide levels >100 pg/ml, right ventricular outflow and peak tricuspid regurgitant pressure gradient exceeding 30 mmHg on echocardiography, exclusion of pulmonary thromboembolism by high-resolution computed tomography or pulmonary scintigraphy, and no deteriorated lung fibrosis that could cause pulmonary hypertension.

### Diagnosis of persistent digital ischemia

Each of the 16 patients in this category had persistent cyanotic lesions on the digits, present for more than 2 years at the time of the present study, and a history of or existing digital ulcers or necrosis. The radial artery pulse in each patient was normal, and atherosclerotic ischemia was excluded clinically. Three of the 16 patients also had PAH.

### Control patients

Serum samples from 24 CTD patients who had neither PAH nor persistent digital ischemia were collected randomly (that is, independent of the activity of their CTD) on the ward or in the outpatient clinic. These samples served as disease controls.

### Normal control subjects

Serum samples were obtained from 28 healthy volunteers (23 females and five males) who were free from any active or chronic diseases, including hypertension. Before bleeding, each subject was confirmed to have normal blood pressure in the arms. The mean age of the 28 subjects was 32.0 ± 9.5 years.

### Detection of anti-ACE2 antibodies in patient serum

We established an ELISA using purified recombinant human ACE2. A plasmid DNA-encoding human ACE2 cDNA, which was kindly gifted from Dr Hyeryun Choe (Harvard Medical School, Cambridge, MA, USA), was introduced into 293 free-style cells, according to the manufacturer's protocol (Invitrogen, Carlsbad, CA, USA). Culture supernatant was harvested on day 2 after transfection, dialyzed against 25 mM Tris-Cl (pH 8.5), and applied onto a DEAE column. ACE2 was eluted with 250 mM NaCl, and was dialyzed against for further use. About 12.5 μg/ml recombinant human ACE2 were first coated overnight onto a 96-well plate with bicarbonate buffer (pH 9.6) at 4°C. The wells were then treated with a blocking buffer composed of 5% BSA in PBS and washed with buffer composed of 20 mM Tris-HCl (pH 7.5), 150 mM NaCl, and 0.1% Tween 20. The sera of the patients and normal healthy volunteers were added to the plate and incubated for 1 hour at room temperature. Bound ACE2 antibodies were detected using horseradish peroxidase-conjugated anti-human IgG antibody. The optical density at 450 nm was measured after a 30-minute incubation with SureBlue TMB microwell peroxidase substrate (Kirkegaard & Perry Laboratories Inc., Gaithersburg, MD, USA). All samples were analyzed in triplicate, and the values were normalized with the positive control.

### Measurement of serum ACE2 activity

Zinc-dependent ACE2 enzymatic activity was measured according to the methods described previously [14,15]. Briefly, 20 μM (7-methoxycoumarin-4-yl) acetyl-APK-(2,4-dinitrophenyl)-OH (amino acids depicted by single letters) (AnaSpec Inc., Fremont, CA, USA) - a fluorogenic substrate [14] - was incubated with 1 μl test serum. Fluorescence was monitored using Safire2 (TECAN, Männedorf, Switzerland) at the excitation and emission wavelengths of 320 nm and 450 nm, respectively.

Preliminary experiments revealed that the relative fluorescence unit (RFU) value was not increased when the substrate was simply incubated without the enzyme (data not shown). Among 20 healthy humans, two samples showed significantly high autofluorescence - in these cases, the RFU values decreased during the incubation even with the substrate. We excluded these samples because it was not possible to evaluate the ACE2 enzyme activity.

The RFU values obtained by the APK substrate were almost completely inhibited by an ACE2 inhibitor, DX600 (Phoenix Pharmaceuticals Inc., Burlingame, CA, USA) (Additional file 1). Additionally, the RFU values were statistically significant at both 60 and 70 minutes of the incubation (Additional file 2). Based on these observations, we used the RFU values at 70 minutes without subtracting the residual RFU counts resistant to DX600 [15]. The relative ACE2 activity (%) in each sample was further calculated based on the level of ACE2 activity in a mixture of serum samples from 28 healthy subjects (a reference serum). The assay was performed in triplicate and the mean values of three independent measurements of the ACE2 ELISA and ACE2 activity assay were used for the statistical analysis by Student's *t *test.

### Detection of serum ACE2 protein

An anti-human ACE2 goat polyclonal antibody (R&D Systems, Minneapolis, MN, USA) was used for immunoprecipitation. Serum was first treated for 15 minutes with 100 mM MgCl_2_, 70 units DNase I (Takara Bio Inc., Shiga, Japan) and 500 μg/ml Ribonuclease A (Sigma, St Louis, MO, USA) at room temperature, and was then incubated with anti-human ACE2 antibody at 4°C for 2 hours. After incubation, the immune complexes were recovered using Protein G Sepharose™ 4 Fast Flow (GE Healthcare, Amersham, Buckinghamshire, UK) and subjected to SDS-PAGE, followed by western blot analysis. To detect ACE2 proteins, an anti-human ACE2 mouse monoclonal antibody was used (R&D Systems).

### Purification of IgG from serum

Five microliters of serum were incubated with Protein G Sepharose™ 4 Fast Flow in 20 mM sodium phosphate buffer (pH 7.0) for 2 hours at 4°C. The immune complexes were then washed and the IgG-bound beads eluted with 0.1 M glycine-HCl (pH 2.7). The recovered IgG was immediately neutralized with 1 M Tris-HCl (pH 8.0). IgG was detected by SDS-PAGE followed by Coomassie Brilliant Blue staining. IgG fractions were prepared from three vasculopathy patients and three healthy subjects. The effect of each IgG on the activity of recombinant human ACE2 (standard rACE2; R&D Systems) was examined as described.

## Results

### Disease status of patients at the time of the present study

The demographics of patients with CTD and PAH or persistent digital ischemia (vasculopathy patients, n = 18) and of the control CTD patients (n = 24) are presented in Table 1. The control patients had neither manifestations nor a history of these vasculopathies.

**Table 1:** Patient demographics

Parameter	Vasculopathy patients	Control patients
Number of patients	18 (17 females, 1 male)	24 (21 females, 3 males)
Age (years)	50.5 ± 14.3	46.8 ± 17.8
Disease entities	SLE (4), SSc (6), MCTD (8)	SLE (21), SSc (2), MCTD (1)
Constrictive vasculopathies	PAH (5); all patients had SSc^a^; persistent digital ischemia (16)	None

Data presented as n or mean ± standard deviation. PAH, pulmonary arterial hypertension; MCTD, mixed connective tissue disease; SLE, systemic lupus erythematosus; SSc, systemic sclerosis. ^a^Three patients also had digital ulcers; of these, one patient had a history of scleroderma renal crisis.

#### Vasculopathy patients

Of the 18 vasculopathy patients, three (Patients 1, 5, and 12) were hospitalized for treatment of an exacerbation of their CTD. Patient 1, with SLE, had fever, pancytopenia, and progressing digital necrosis of the fingers and toes. Patient 5, with the diffuse form of SSc, was referred to our hospital and admitted because of heart failure caused by severe PAH of recent origin. Despite high-dose steroid therapy for PAH and conventional therapy for heart failure, she died of sudden cardiac arrest. Patient 12, with SLE, had fever, pancytopenia, diffuse erythema, and painful digital ischemia accompanied by chilblain lupus.

The remaining 15 outpatients (all had persistent digital ischemia and four also had PAH) were being treated with prostacyclins or calcium antagonists without apparent improvement, and all of the patients with digital ischemia had suffered from digital ulcers or digital necrosis within 2 years of the present study. In the five patients with PAH, the mean ± standard deviation peak tricuspid regurgitant pressure gradient was 51 ± 13 mmHg (range, 39 to 70 mmHg; median, 50 mmHg) on echocardiography within 6 months of the present study, and the mean plasma brain natriuretic peptide level was 639 ± 623 pg/ml (range, 107 to 1,312 pg/ml; median, 568 pg/ml) at the time of this study. Systemic hypertension was observed in four SLE patients and in one SSc patient. Three of the patients had been treated with a calcium antagonist or ACE inhibitor for years, and hypertension developed in the remaining two patients (Patients 1 and 12) during steroid therapy at the time of study.

#### Control connective tissue disease patients

Of these 24 patients, 11 were hospitalized: nine patients for exacerbated SLE (six with lupus nephritis, two with neuropsychiatric lupus, and one with thrombocytopenia), one SLE patient for aseptic bone necrosis, and one SSc patient for accidental cholecystitis. The remaining 13 outpatients had stable disease on a maintenance dose of steroid therapy. Recent Raynaud's phenomena were observed in two SLE patients, two SSc patients, and one MCTD patient. Systemic hypertension was observed in one SLE patient, who had been treated with an Ang II receptor blocker.

### ELISA for serum anti-ACE2 antibodies

**Figure 1:**
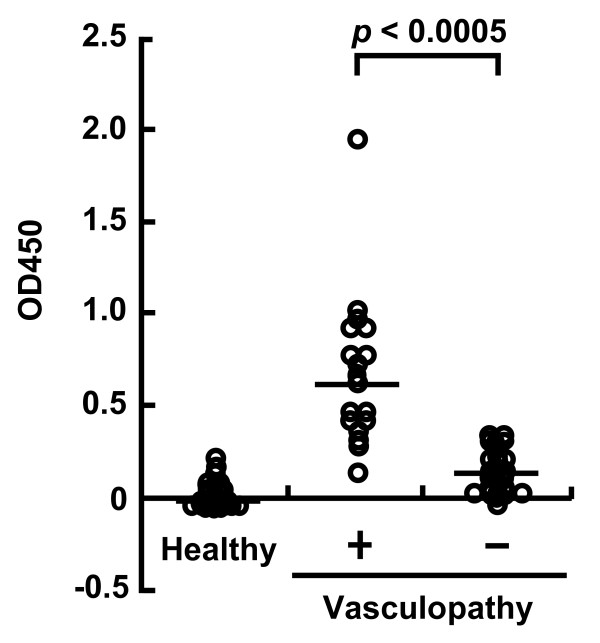
**Summarized results of ELISA for detecting serum anti-angiotensin-converting enzyme 2 antibodies**. The ELISA scores of the vasculopathy patients (n = 18) are significantly higher than those of patients without vasculopathy (n = 24) and those of healthy subjects (n = 28). Bars indicate the median. *P *< 0.01.

All of the patients had received a maintenance dose of steroid therapy by the time of blood sampling. Serum samples were obtained from inpatients just before starting additional immunosuppressive therapy for a disease flare-up, and from outpatients. Sera from 17 of the 18 (94%) vasculopathy patients showed reactivity to ACE2 with ELISA values above the baseline level value, which was determined as the mean ELISA score in the 28 normal control subjects. The mean ELISA score was significantly higher in the vasculopathy patients than in the control patients (Figure 1). These results suggest that anti-ACE2 antibodies in the serum are associated with constrictive vasculopathies, PAH, or persistent digital ischemia.

### Inhibition of ACE2 activity by IgG purified from patient serum

**Figure 2:**
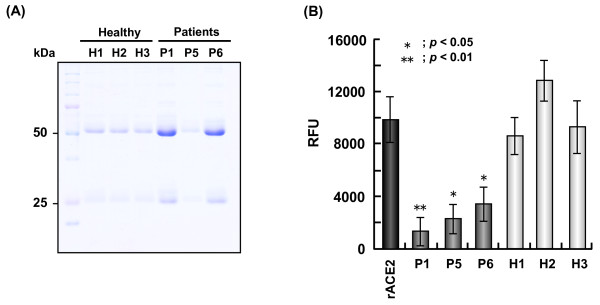
**Inhibition of angiotensin-converting enzyme 2 activity by IgG purified from patient serum**. **(a) **Purified IgG from the sera of healthy volunteers (H1 to H3) and patients with vasculopathy (P1, P5, and P6) was detected by SDS-PAGE and Coomassie Brilliant Blue staining. The molecular weights of the heavy (50 kDa) and light (25 kDa) chains of IgG are shown. **(b) **The inhibition of angiotensin-converting enzyme (ACE) 2 activity by 5 μg purified IgG was examined in triplicate assays. As a control, ACE2 activity in standard rACE2 was measured in the absence of IgG. As shown, ACE2 activity was significantly reduced when the recombinant enzyme was co-incubated with IgG from the patients. *P *< 0.01. RFU, relative fluorescence unit.

IgG fractions from the sera of three healthy subjects and three vasculopathy patients (Patients 1, 5, and 6) were prepared (Figure 2a). The activity of standard rACE2 was measured in 5 μg aliquots. The IgG fractions from the vasculopathy patients, but not from the healthy subjects, suppressed the rACE2 enzyme activity significantly compared with that in the absence of IgG (Figure 2b).

### Serum ACE2 activity in vasculopathy patients, control patients, and healthy subjects

First, the RFU value of the ACE2 enzyme activity, which was blocked by DX600 (Additional file 1), was determined in 18 patients with vasculopathy, in 16 control patients without vasculopathy, and in 26 healthy subjects. The relative rate of serum ACE2 enzyme activity was then compared with a reference mixture composed of sera from healthy subjects. Serum ACE2 activity could not be measured in one control patient and two healthy subjects due to high fluorescence at the start of incubation, and sera from the remaining seven control patients were not available for these experiments. The mean ± standard deviation relative ACE2 activity was significantly lower in the vasculopathy patients (64.8 ± 16.4, n = 18) compared with that in the healthy subjects (86.6 ± 32.2, n = 26, *P *= 0.0055) and control patients (165.1 ± 99.1, n = 16, *P *= 0.0010).

**Figure 3:**
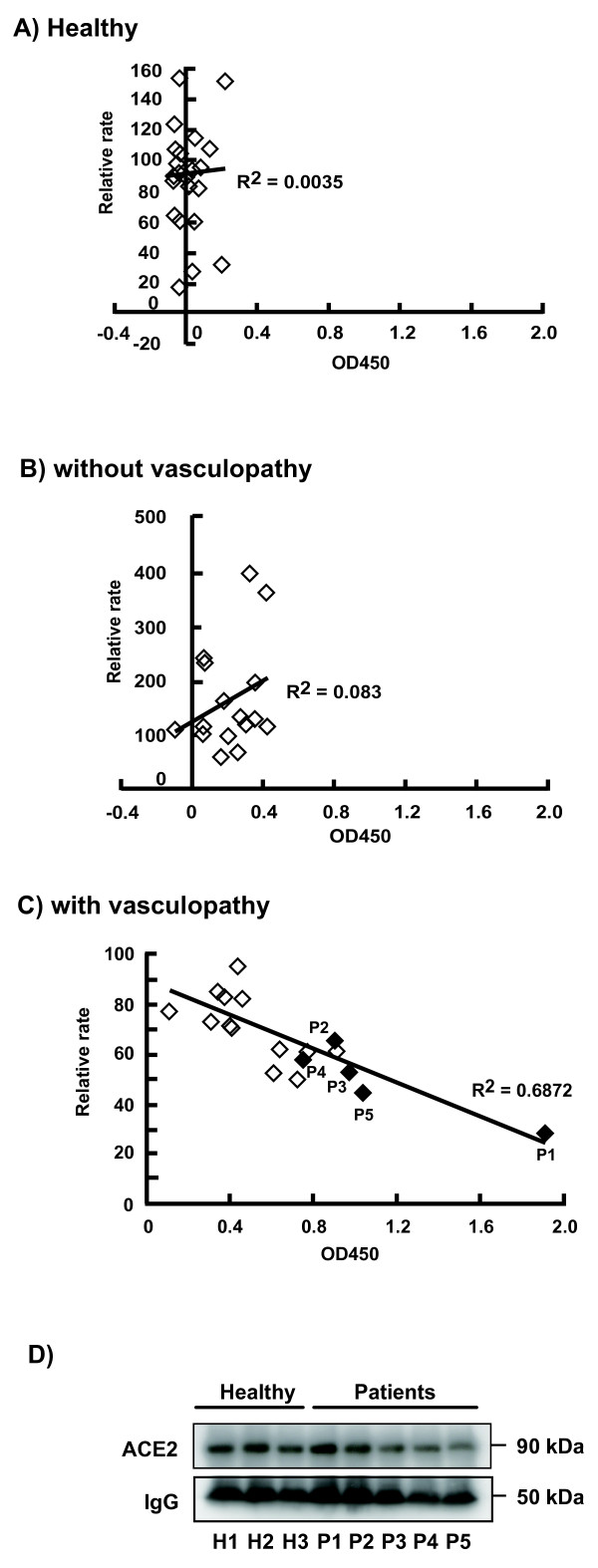
**Inverse correlation between the presence of angiotensin-converting enzyme 2 autoantibodies and activity in vasculopathy patients**. The relative (%) activity of serum angiotensin-converting enzyme (ACE) 2 compared with the reference value and ELISA score of the same sera were plotted in **(a) **26 healthy subjects, **(b) **16 patients without vasculopathy, and **(c) **18 vasculopathy patients, respectively, and Pearson correlation coefficients were calculated. The relative ACE2 activities were determined based on a reference serum (mixed sera from healthy subjects). **(d) **Western blot analysis to detect ACE2 protein in serum. Immunoprecipitation followed by immunoblotting was performed on sera of healthy subjects (H1 to H3) and vasculopathy patients (P1 to P5). ACE2 was detected as a major band about 90 kDa in size (see also Additional file 3).

To clarify the correlation between ACE2 activity and the presence of autoantibodies to ACE2, the ELISA scores for serum anti-ACE2 antibodies and serum ACE2 activity were plotted (Figure 3). Our data indicate that the titers of anti-ACE2 antibodies were inversely proportional to ACE2 peptidase activity in the vasculopathy patients (Figure 3c). In contrast, no correlation was observed in the healthy subjects or control patients, suggesting that the low serum ACE2 activities in most of the vasculopathy patients were due to the presence of anti-ACE2 antibodies. To assess ACE2 expression in the vasculopathy patients, we tested for ACE2 by immunoprecipitation followed by immunoblotting. As shown in Figure 3d, the ACE2 levels in Patients 1 and 5, who were deficient in ACE2 activity (Figure 3c), were comparable with those in three healthy subjects, clearly indicating that the decreased ACE2 activity in the vasculopathy patients was due to a functional deficiency. The relative ACE2 protein levels in the patients are shown in Additional file 3. The signal intensities of ACE2 protein and the IgG heavy chain shown in Figure 3d were measured and normalized.

### Recovery of serum ACE2 activity after therapy in one patient

**Figure 4:**
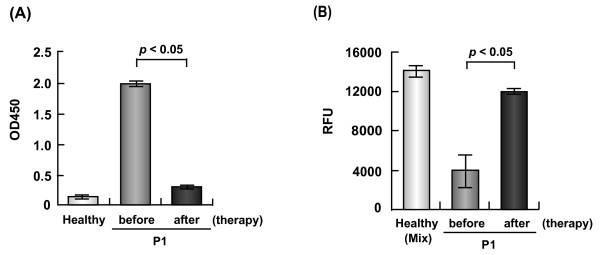
**Anti-angiotensin-converting enzyme 2 antibodies and activity in a systemic lupus erythematosus patient**. Anti-angiotensin-converting enzyme (ACE) 2 antibodies and ACE activity in a systemic lupus erythematosus patient (SLE) before and after therapy. **(a) **The ELISA score and **(b) **the ACE2 activity of Patient 1 (P1) recovered significantly after therapy.

The exacerbated SLE and progressive necrosis of the fingers and toes in Patient 1, a 25-year-old female, were treated with intravenous methylprednisolone at a dose of 500 mg/day for 3 days, followed by 30 mg/day oral prednisolone combined with three sessions of double-filtration plasmapheresis and conventional therapy, including prostanoids, stellate ganglion anesthesia, and local care. Her cutaneous lesions healed completely before discharge, without amputation of any digits. Serum tests 2 months after the start of therapeutic intervention showed that the ELISA score for anti-ACE2 antibodies was reduced markedly (*P *< 0.05) (Figure 4a) and the ACE2 activity had recovered significantly (*P *< 0.05) (Figure 4b), compared with the values before therapy. An ELISA for anti-ACE2 antibodies in the waste double-filtration plasmapheresis fluid was negative, and thus we could not evaluate whether double-filtration plasmapheresis had removed these antibodies from the patient's blood. The anti-ACE2 antibodies were probably decreased as a result of immunosuppressive therapy using high-dose pulse steroids.

The other two vasculopathy inpatients (Patients 5 and 12) also received immunosuppressive therapy during the present study and had high ELISA scores for anti-ACE2 antibodies before therapy. The serum of Patient 5 was not studied after therapy, because she died suddenly. For Patient 12, the ELISA scores before therapy and after 2 months of therapy with 30 mg/day prednisolone were 0.68 ± 0.02 and 0.36 ± 0.01, respectively. These results were not significantly different, and the titer tended to decrease after therapy. The relative rates (%) of serum ACE2 activity in this patient before and after therapy were 52.2 ± 19.1 and 63.1 ± 4.8 (*P *= NS), based on the level of activity detected in the reference sample, respectively.

## Discussion

Persistent digital ischemia and clinically evident PAH are intractable and relatively infrequent complications of SLE, SSc, and MCTD - although latent forms of vasculopathies, including sporadic abnormalities such as Raynaud's phenomena, may be more prevalent in CTD patients. Immunosuppressive therapy can improve PAH in some patients with SLE, SSc, or MCTD [16], and in most SLE patients [17] via an unknown therapeutic mechanism. The treatment of digital ischemia remains an unsolved clinical problem for physicians. Our study suggests, for the first time, that autoantibodies to ACE2 are present in the serum of patients with persistent constrictive vasculopathies. Furthermore, the elevated ELISA scores for anti-ACE2 antibodies correlated with reduced ACE2 activity. The serum autoantibodies to ACE2 may therefore inhibit ACE2 activity, and the inhibition may be reversible after immunosuppressive therapy, based on the data from Patient 1. There were small numbers of patients with systemic hypertension in both the vasculopathy and control patient groups. We could not determine whether their hypertension was essential hypertension, or was caused by steroid therapy or ACE2 inhibition.

The inhibition of ACE2 by autoantibodies may result in reduced physiological levels of the vasoprotective agent Ang(1-7) in the local vascular milieu, which may induce vasculopathies in patients with underlying disease such as CTD. Vasodilating drugs - including prostanoids, endothelin-receptor antagonists, and phosphodiesterase type-5 inhibitors - have been used widely to treat PAH and have been partially effective for some PAH patients [18]. No effective drugs have been found for persistent digital ischemia [19]. Our study suggests that ACE2 activation or administration in experimental PAH [5,6] may be applicable as a therapy for PAH or persistent digital ischemia in patients with CTD.

## Conclusions

Anti-ACE2 autoantibodies may be associated with constrictive vasculopathy in patients with connective tissue disease.

## Abbreviations

ACE: angiotensin-converting enzyme; Ang: angiotensin; BSA: bovine serum albumin; CTD: connective tissue disease; ELISA: enzyme-linked immunosorbent assay; MCTD: mixed connective tissue disease; PAH: pulmonary arterial hypertension; PBS: phosphate-buffered saline; rACE2: recombinant human angiotensin-converting enzyme 2; RFU: relative fluorescence unit; SLE: systemic lupus erythematosus; SSc: systemic sclerosis.

## Competing interests

The authors declare that they have no competing interests.

## Authors' contributions

YT and SH equally contributed to this study. YT performed the ELISA, clinical practice, and wrote the paper. SH prepared a recombinant ACE2 protein, carried out the assays for ACE2 activities, and wrote the methods. YI designed the experiments. AM designed the research and completed the paper.

## Supplementary Material

Additional file 1 **Inhibition of the enzyme activity by an ACE2 inhibitor**. By preincubation with DX600 for 30 minutes, the enzyme activity was almost completely blocked (*P *< 0.01). H, healthy volunteer; P, patients with vasculopathy; CP, control patients without vasculopathy. The activity was also blocked by the addition of ethylenediamine tetraacetic acid (EDTA) (data not shown), indicating that the activity would depend on the presence of zinc ion. Click here for file

Additional file 2 **Optimization of the measurement analysis of ACE2 activity**. ACE2 activity was measured chronologically with the fluorogenic substrate and plotted for 90 min. The relative fluorescence unit (RFU) values increased within 70 minutes and then declined. Each plot is a representative result of three independent experiments. In each experiment, a rACE2 (a standard) and a reference serum (a mixture of sera from 28 healthy subjects) were assayed simultaneously with sample sera from healthy subjects, control patients, or vasculopathy patients. The difference of the ACE2 enzyme activity between healthy subjects and vasculopathy patients was statistically significant (*P *< 0.01) both at 60 and 70 minutes of the incubation. Click here for file

Additional file 3 **Relative intensity of the ACE2 protein levels in patients**. The signal intensities of ACE2 protein and the IgG heavy chain shown in Figure 3d were measured and normalized. Each relative intensity was standardized with that of sample H1. Click here for file
