# Supplementary figures and images for: Autoantibodies to angiotensin-converting enzyme 2 in patients with connective tissue diseases
Source: Arthritis Res Ther. 2010 May 14;12(3):R85. doi: 10.1186/ar3012 (PMC2911869; doi:10.1186/ar3012)

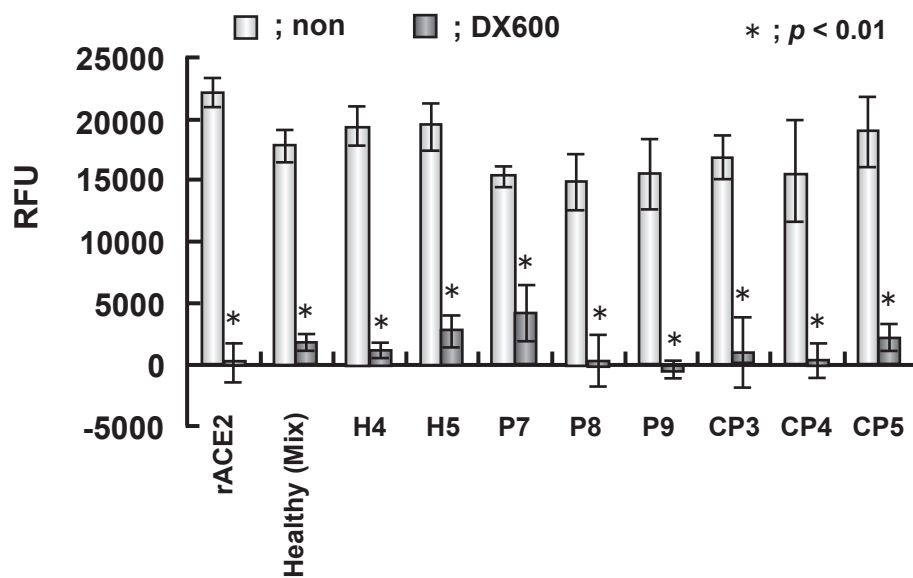

Supplement: Additional file 1 — Inhibition of the enzyme activity by an ACE2 inhibitor. By preincubation with DX600 for 30 minutes, the enzyme activity was almost completely blocked (P < 0.01). H, healthy volunteer; P, patients with vasculopathy; CP, control patients without vasculopathy. The activity was also blocked by the addition of ethylenediamine tetraacetic acid (EDTA) (data not shown), indicating that the activity would depend on the presence of zinc ion. [file ar3012-S1.PDF]

### A) Healthy

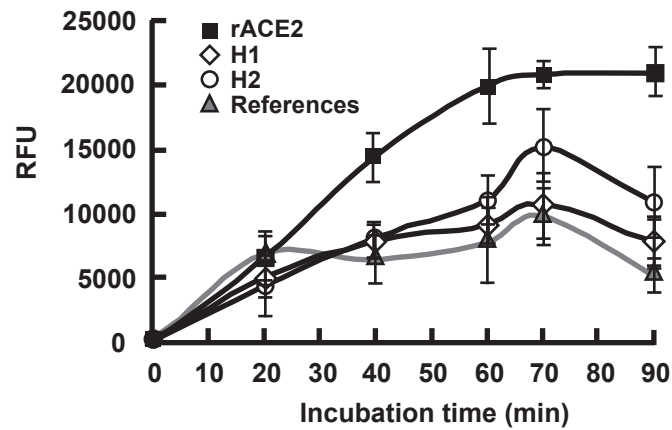

### B) with vasculopathy

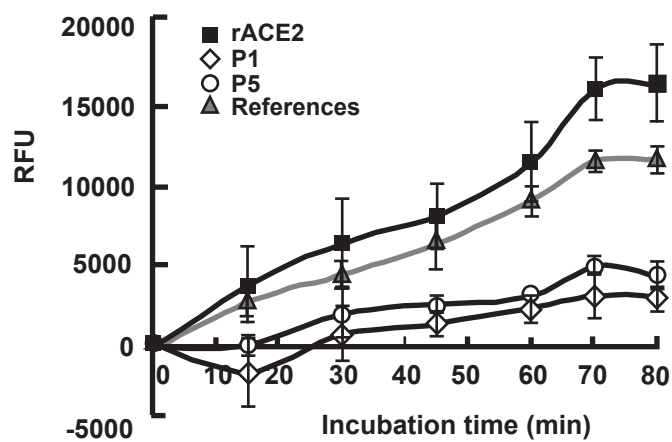

### C) without vasculopathy

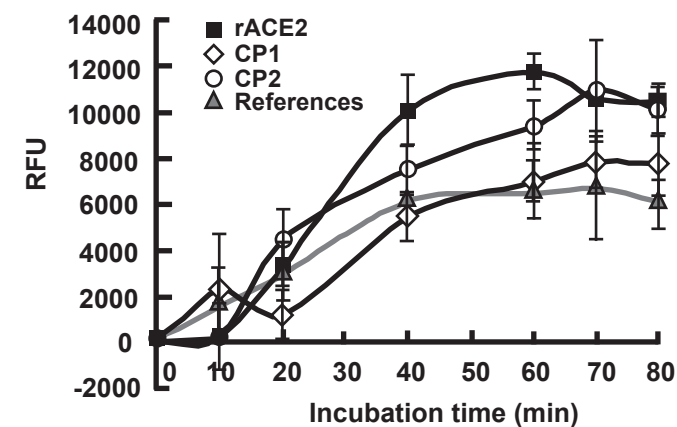

Supplement: Additional file 2 — Optimization of the measurement analysis of ACE2 activity. ACE2 activity was measured chronologically with the fluorogenic substrate and plotted for 90 min. The relative fluorescence unit (RFU) values increased within 70 minutes and then declined. Each plot is a representative result of three independent experiments. In each experiment, a rACE2 (a standard) and a reference serum (a mixture of sera from 28 healthy subjects) were assayed simultaneously with sample sera from healthy subjects, control patients, or vasculopathy patients. The difference of the ACE2 enzyme activity between healthy subjects and vasculopathy patients was statistically significant (P < 0.01) both at 60 and 70 minutes of the incubation. [file ar3012-S2.PDF]

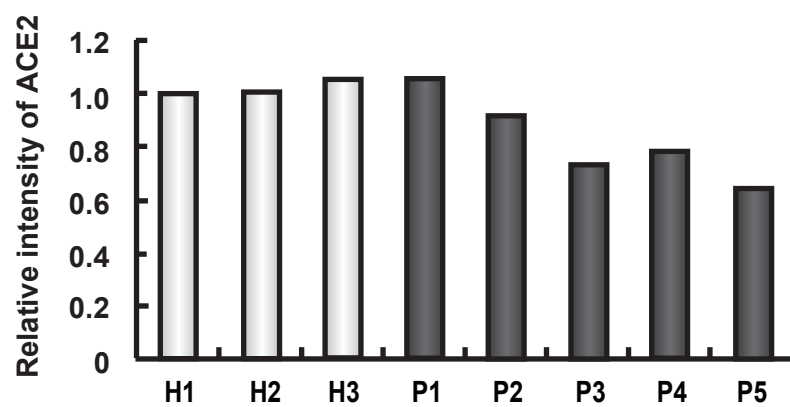

Supplement: Additional file 3 — Relative intensity of the ACE2 protein levels in patients. The signal intensities of ACE2 protein and the IgG heavy chain shown in Figure 3d were measured and normalized. Each relative intensity was standardized with that of sample H1. [file ar3012-S3.PDF]
